# Detection and geographic distribution of seven facultative endosymbionts in two *Rhopalosiphum* aphid species

**DOI:** 10.1002/mbo3.817

**Published:** 2019-03-25

**Authors:** Jianqing Guo, Xuewei Liu, Nicolas Poncelet, Kanglai He, Frédéric Francis, Zhenying Wang

**Affiliations:** ^1^ State Key Laboratory for Biology of Plant Diseases and Insect Pests Institute of Plant Protection Chinese Academy of Agricultural Sciences Beijing China; ^2^ Functional and Evolutionary Entomology Gembloux Agro‐Bio Tech University of Liège Gembloux Belgium; ^3^ College of Agriculture and Forestry Hebei North University Zhangjiakou China

**Keywords:** cereal aphid, China, Europe, facultative endosymbiont, infection, *Rhopalosiphum maidis*, *Rhopalosiphum padi*

## Abstract

Study of the mutualistic associations between facultative symbionts and aphids are developed only in a few models. That survey on the situation and distribution of the symbionts in a certain area is helpful to obtain clues for the acquisition and spread of them as well as their roles played in host evolution. To understand the infection patterns of seven facultative symbionts (*Serratia symbiotica*,* Hamiltonella defensa*,* Regiella insecticola*,* Rickettsia*,* Spiroplasma*,* Wolbachia,* and *Arsenophonus*) in *Rhopalosiphum padi* (Linnaeus) and *Rhopalosiphum maidis* (Fitch), we collected 882 *R. maidis* samples (37 geographical populations) from China and 585 *R. padi* samples (32 geographical populations) from China and Europe. Results showed that both species were widely infected with various symbionts and totally 50.8% of *R. maidis* and 50.1% of *R. padi* were multi‐infected with targeted symbionts. However, very few *Rhopalosiphum* aphids were infected with *S. symbiotica*. The infection frequencies of some symbionts were related to the latitude of collecting sites, suggesting the importance of environmental factors in shaping the geographic distribution of facultative symbionts. Also, *R. maidis* and *R. padi* were infected with different *H. defensa* strains based on phylogenetic analysis which may be determined by host ×symbiont genotype interactions. According to our results, the ubiquitous symbionts may play important roles in the evolution of their host aphid and their impacts on adaptation of *R. padi* and *R. maidis* were discussed as well.

## 1 INTRODUCTION

The symbiotic relationship between aphids and endosymbionts is ubiquitous. To date, the endosymbionts harbored by aphids are divided into obligate (or primary) and facultative (or secondary) symbionts. Obligate symbiont *Buchnera aphidicola* is indispensable for aphids since it can offer essential amino acids that the aphid host cannot synthesize themselves or obtain from the phloem of plants (Baumann, 2005; Douglas, 1998) while facultative symbionts are not strictly required for host survival and reproduction (Oliver, Degnan, Burke, & Moran, 2010). However, recent research found that one strain of *Serratia symbiotica* in *Cinara tujafilina* has been undergoing the transformation from facultative symbiont to become an obligate intracellular one (Manzano‐Marín & Latorre, 2014) and *Wolbachia* has evolved to become a co‐obligitory symbiont in the banana aphid *Pentalonia nigronervosa* (De Clerck et al., 2015). Nevertheless, the facultative symbionts do confer traits which impact on host aphid fitness (Guo et al., 2017). A key trait conferred by symbionts is resistance, this “symbiont‐mediated resistance” concept was first proposed by Oliver, Moran, and Hunter (2005) and states that secondary symbionts can confer host aphid defense toward adverse situation. For instance, *S. symbiotica* can confer heat resistance for host aphid (Gómez‐Valero et al., 2004; Montllor, Maxmen, & Purcell, 2002) and *Hamiltonella defensa* can protect host aphids such as pea aphid *Acyrthosiphon pisum* (Łukasik, van Asch, Guo, Ferrari, & Godfray, 2013; Oliver, Campos, Moran, & Hunter, 2008; Oliver, Russell, Moran, & Hunter, 2003; Oliver et al., 2005), *Sitobion avenae* (Łukasik, Dawid, Ferrari, & Godfray, 2013), *Rhopalosiphum padi* (Linnaeus) (Leybourne, Bos, Valentine, & Karley, 2018), and *Aphis craccivora* (Asplen et al., 2014) against parasitoid wasps. Moreover, the impacts of nine facultative symbionts on aphids were described one by one (Guo et al., 2017) and the global geographic distribution of eight facultative symbionts in aphids tested so far was summarized by Zytynska and Weisser (2016).

Facultative symbionts are generally inherited maternally with high frequencies (Luan, Sun, Fei, & Douglas, 2018; Luan et al., 2016), however, horizontal transmission of facultative symbionts occurs occasionally (Russell, Latorre, Sabater‐Muñoz, Moya, & Moran, 2003; Sandström, Russell, White, & Moran, 2001). Despite the horizontal transmission and substantial benefits conferred by facultative symbionts, the bacteria are still maintained at intermediate level in nature (Castañeda, Sandrock, & Vorburger, 2010; Henry, Maiden, Ferrari, & Godfray, 2015; Unckless & Jaenike, 2012; Watts, Haselkorn, Moran, & Markow, 2009; Zytynska & Weisser, 2016). Also, the infection frequencies are dynamic, differing across temporal and spatial gradients, and food‐plant associations (Oliver, Smith, & Russell, 2014). Most researchers agree with the idea that there exist costs for hosts to harbor the facultative symbionts (Oliver et al., 2008; Scarborough, Ferrari, & Godfray, 2005) and fitness reduction in aphids containing the facultative symbionts have been found in some cases (Laughton, Fan, & Gerardo, 2013; Vorburger & Gouskov, 2011) such as the infection of *H. defensa* could reduce aphid longevity (Vorburger & Gouskov, 2011). However, multiple infections of facultative symbionts are common in nature (Ferrari, West, Via, & Godfray, 2012; Oliver et al., 2014; Russell et al., 2013). The interactions between different symbionts coaffecting the host are complex. Some symbionts exhibit additive effects to the host: coinfection of *S. symbiotica* and *H. defensa* in *A. pisum* resulted in higher resistance to parasitism of *Aphidius ervi* (Oliver, Moran, & Hunter, 2006). However, inhibiting effects were found in another case: *A. pisum* coinfected with *Rickettsiella viridis* and *H. defensa* were more exposed to predation (Polin, Le Gallic, Simon, Tsuchida, & Outreman, 2015).

Both *Rhopalosiphum maidis* (Fitch) and *R. padi* are two important pest species on maize especially during the later growth stage, sharing the same niche, feeding on leaves, leaf sheath, husks of maize. What's more, both *Rhopalosiphum* species can transmit viruses including Maize dwarf mosaic virus and Barley yellow dwarf virus (Chen et al., 1996; Parry, Macfadyen, & Kriticos, 2012; Saksena, Singh, & Sill, 1964; Smyrnioudis, Harrington, Clark, & Katis, 2001) which may cause serious economic damages to their host plants. Recent research showed the importance of facultative symbionts for host aphids such as *A. pisum* (Łukasik, van Asch, et al., 2013), *A. craccivora* (Wagner et al., 2015), *Aphis fabae* (Castañeda et al., 2010), and *R. padi* (Leybourne et al., 2018). Several studies have assessed endosymbiont infections in *R. padi* to date. For instance, *H. defensa*‐infected nymphs of *R. padi* collected from UK showed fivefold higher resistance to the parasitoid wasp *Aphidius colemani* (Viereck) than uninfected nymphs (Leybourne et al., 2018). De la Peña, Vandomme, and Frago (2014) found that *R. padi* collected from northwest of Belgium was only associated with *S. symbiotica,* whereas research showed five *R. padi* individuals collected from wheat harbored SMLS (*Sitobion miscanthi* L. type symbiont) but no *Rickettsia* (Li, Xiao, Xu, Murphy, & Huang, 2011) and an absence of targeted facultative symbionts was found in *R. padi* collected in Chile (Zepeda‐Paulo, Ortiz‐Martínez, Silva, & Lavandero, 2018). However, few research described the infection situation of symbionts in a particular region for *R. maidis* except one which reported that no facultative symbionts were detected from 25 *R. maidis* collected in Morocco (Fakhour et al., 2018). In this study, we conducted an extensive survey of seven facultative symbionts in hosts *R. maidis* and *R. padi* collected from the maize (*Zea mays* L.) in China and four European countries to assess geographic infection patterns of these facultative symbionts.

## 2 MATERIALS AND METHODS

### 2.1 Sample collection

**Figure 1:**
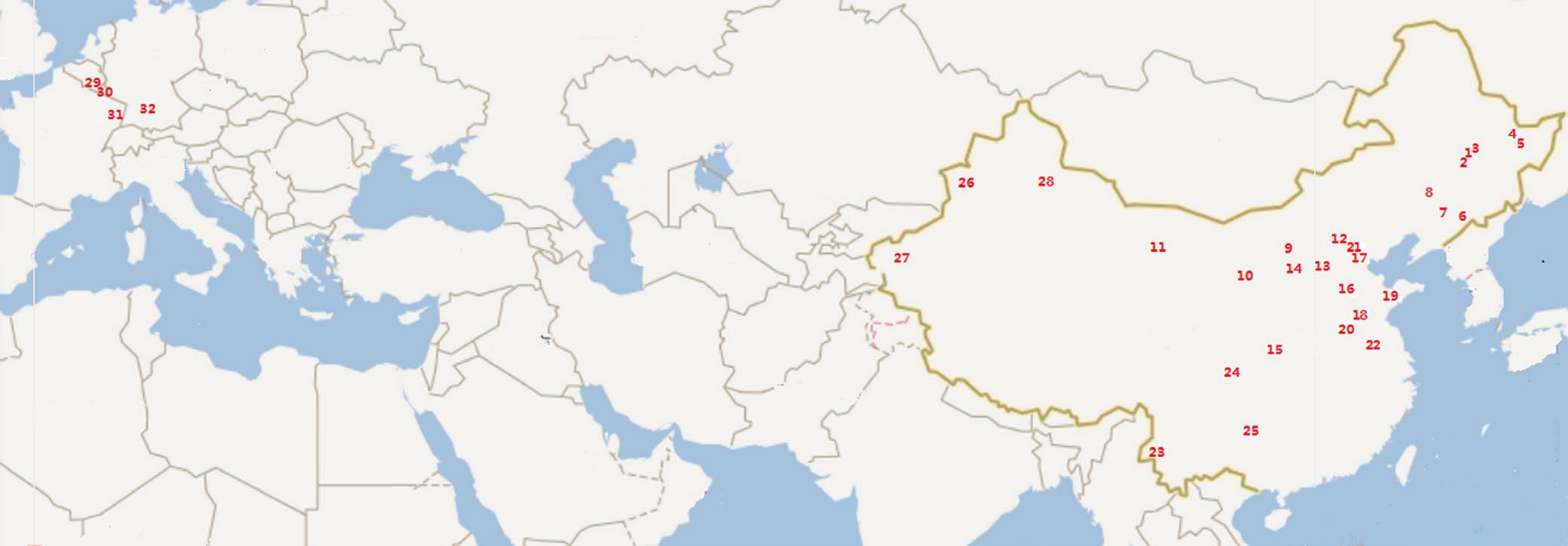
Collecting locations of *Rhopalosiphum padi* in China and Europe. Numbers on the map correspond to locality numbers in Table A1

**Figure 2:**
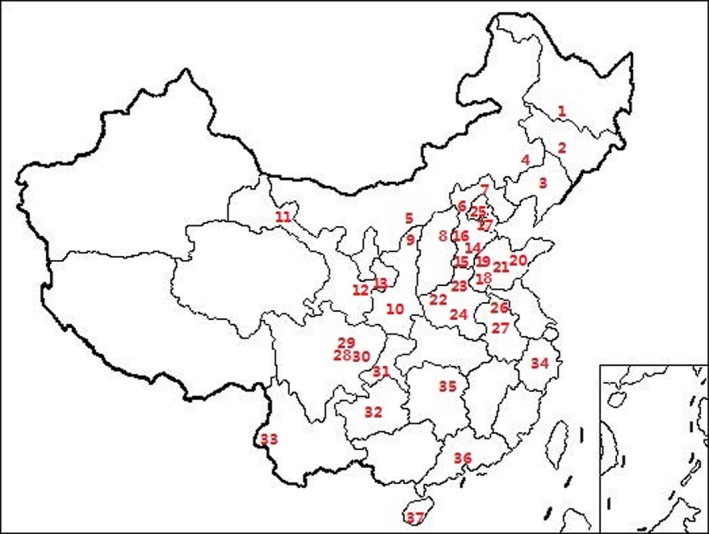
Collecting locations of *Rhopalosiphum maidis* in China. Numbers on the map correspond to locality numbers in Table A2

We collected a total of 882 *R. maidis* from 37 geographical populations and 585 *R. padi* from 32 geographical populations. All aphids were collected from maize and the distance between each two samples was at least 10 m. All these collection sites (except four European populations) were selected to cover the comprehensive maize cultivating areas in China as much as possible and the collection work was done via random generation of co‐ordinates within each site. More than 20 aphids per population were collected for most populations, although some populations may have fewer samples. All samples were identified by COI (mitochondrial cytochrome oxidase I) gene (Primers: LCO1490: 5′‐GGTCAACAAATCATAAAGATATTGG‐3′; HCO2198: 5′‐TAAACTTCAGGGTGACCAAAAAATCA‐3′) (Folmer, Black, Hoeh, Lutz, & Vrijenhoek, 1994) and the information of aphid samples used in this study was listed in Tables A1 and A2 and the collecting locations were labeled on the maps (Figures 1 and 2). All collected aphids were preserved in absolute ethanol and stored at −20°C before molecular analysis.

### 2.2 DNA preparation

Total DNA was extracted from single aphid using TEN buffer (10 mM Tris–HCl pH = 8, 2 mM EDTA pH = 8, 0.4 M NaCl), 20% SDS and 5 M NaCl solution according to the salting‐out method (Sunnucks & Hales, 1996). 20–30 μl TE buffer was used to dissolve the DNA precipitate and the DNA quality was assessed with a Nanodrop 2000/2000C instrument. Then the DNA samples were kept at −20°C for further use.

### 2.3 Symbionts detection

All 1,467 samples of the two aphid species were screened for the presence of seven facultative symbionts. Diagnostic PCR analysis was conducted using the specific primers listed in Table A3 to detect respective endosymbionts. PCR reactions of 20 μl volume for each sample were carried out under the following conditions: an initial denaturation at 94°C for 4 min, followed by 35 cycles of 94°C for 30 s, 55°C for 30 s, and 72°C for 30 s, and a final extension at 72°C for 5 min. DNA from aphids in laboratory of functional and evolutionary entomology (University of Liège) known to harbor a specific symbiont was used as a positive control and solution without DNA template was used as a negative control. The PCR products were detected by 1.5% agarose gel electrophoresis.

### 2.4 Sequencing and analysis of *H. defensa*

PCR reactions were performed again in a 50 μl volume with the DNA samples positive with *H. defensa* (*n* = 63 for *R. padi*,* n* = 141 for *R. maidis*), then PCR products were purified using PCR Clean‐up kit (Sangon) and sent for sequencing without cloning. Obtained sequences were verified via BLAST (http://blast.ncbi.nlm.nih.gov/Blast.cgi) and assembled in DNAMAN v6. *H. defensa* sequences downloaded from NCBI (http://www.ncbi.nlm.nih.gov/) of other species were the source for multiple sequence alignment by DNAMAN and MEGA. The phylogenetic analyses were conducted using the Maximum likelihood methods with MEGA 4 software. Clade support was assessed with 1,000 bootstrap replicates (Stamatakis, Hoover, & Rougemont, 2008).

### 2.5 Statistical analysis

Differences in the infection frequency of detected symbionts and the proportion of symbiont number per aphid between *R. padi* and *R. maidis* from 19 common locations (Figures 4 and 5) and between *R. padi* populations from China and Europe (Figures 1 and 6) were determined using two‐tailed Fisher's exact tests implemented in the software SPSS (SPSS v16.0). The linear correlation analysis was accomplished using Pearson distribution with the software SPSS to assess whether the infection frequencies of the symbionts were correlated with the latitudes of collecting sites.

## 3 RESULTS

### 3.1 Seven facultative symbionts were detected in *R. padi* and *R. maidis*

Both *R. maidis* (*n* = 882) and *R. padi* (*n* = 585) were frequently infected with various symbionts (Table 1). The infection frequencies for the seven targeted symbionts varied from 0.2% to 60.9% (Table 1) and only 20.2% of *R. maidis* and 17.1% of *R. padi* were not infected with any of the seven symbionts screened for (Table 2). *Rickettsia* ranked the highest frequency in the two aphid species (51.6% in *R. maidis*; 60.9% in *R. padi*) followed by *R. insecticiola* (34.1% in *R. maidis*; 40.7% in *R. padi*) and *Spiroplasma* (35.8% in *R. maidis*; 26.3% in *R. padi*), whereas both aphids had the lowest infection rate of *S. symbiotica* that only nine samples of *R. maidis* and one sample of *R. padi* were infected.

**Table 1:** Total frequency of detection of each symbiont in the two aphid species

	Proportion of infected aphids (%)						
	*Serratia symbiotica*	*Hamiltonella defensa*	*Regiella insecticola*	*Rickettsia* sp.	*Spiroplasma* sp.	*Wolbachia* sp.	*Arsenophonus* sp.
*Rhopalosiphum maidis*	1.0	16.0	34.1	51.6	35.8	2.8	26.0
*Rhopalosiphum padi*	0.2	10.8	40.7	60.9	26.3	3.3	18.0

**Table 2:** Total frequency of symbiont numbers infected in a single aphid

	Proportion of infected aphids (%)						
	0	1	2	3	4	5	6
*Rhopalosiphum maidis*	20.2	29.0	25.1	17.2	6.2	2.0	0.2
*Rhopalosiphum padi*	17.1	32.8	30.4	13.7	4.6	1.4	0

The trends of symbiont diversity per aphid were similar in both species (Table 2). Aphids infected with only one symbiont ranked the highest proportion of 29.0% in *R. maidis* and 32.8% in *R. padi*, respectively. Totally, around half of the tested aphids were infected with multiple symbionts (50.8% of *R. maidis* and 50.1% of *R. padi*). The double infected samples occupied 25.1% in *R. maidis* and 30.4% in *R. padi*. In addition, two samples of *R. maidis* harbored as many as six facultative symbionts simultaneously and no *R. padi* was infected with six symbionts in a single aphid.

### 3.2 Frequencies of seven facultative symbionts in each population of *R. padi* and *R. maidis*

**Figure 3:**
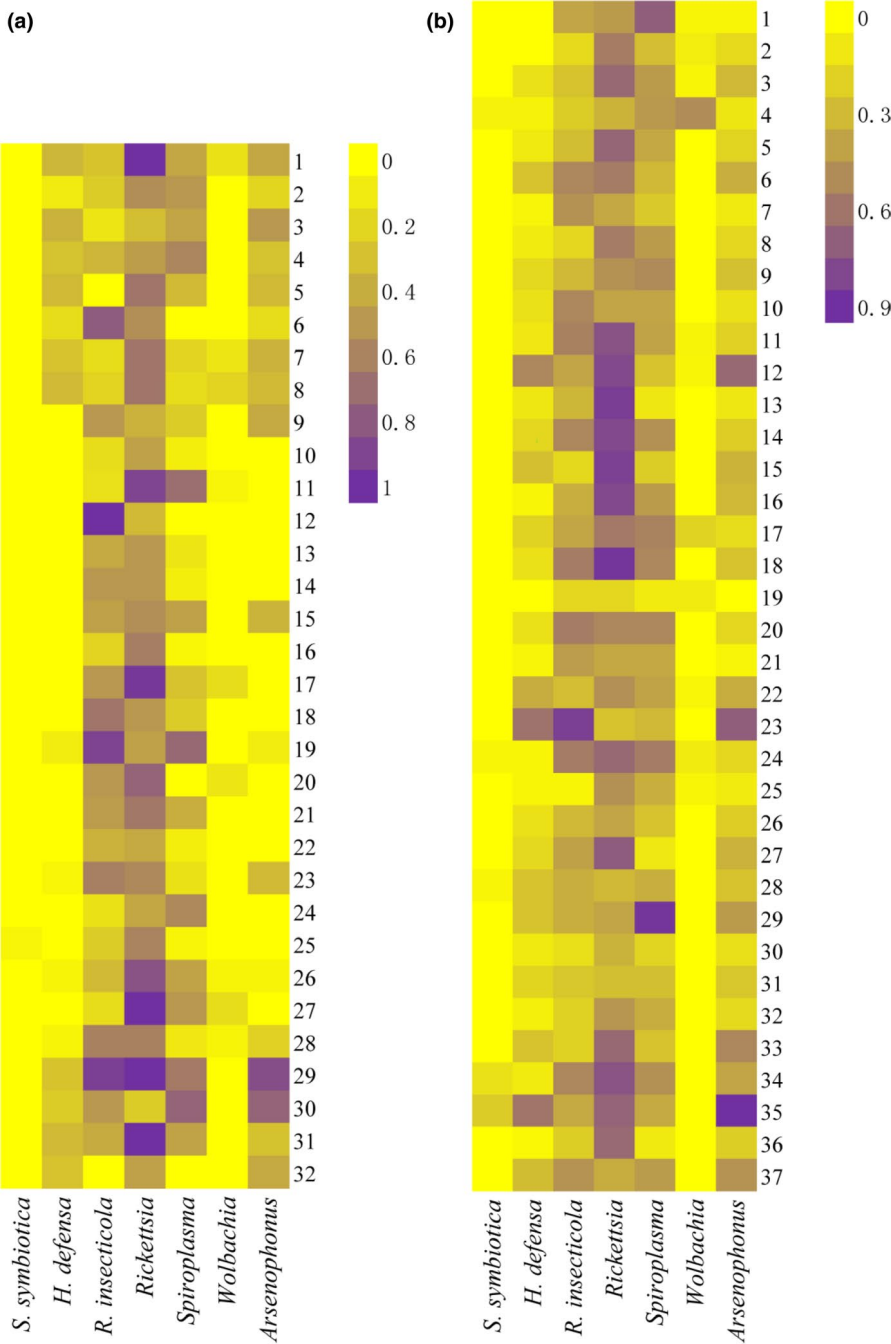
Heatmaps showing proportion of symbiont occurrence in each population. (a) *Rhopalosiphum padi*, (b) *R. maidis*. The infection frequencies of seven facultative symbionts were represented by the values in the heatmaps. Numbers on figure (a) correspond to locality numbers in Table A1 and numbers on figure (b) correspond to locality numbers in Table A2

Two heatmaps displaying the infection frequencies of the symbionts in each population of *R. padi* and *R. maidis* were generated (Figure 3), from which we can found that *Rickettsia*,* R. insecticola* and *Spiroplasma* were found in high densities in both aphid species, whereas *S. symbiotica* was rarely detected. Among all the symbionts, only one population of *R. padi* (GY—5.0% of individuals) and five populations of *R. maidis* (TL—5.3% of individuals, LH—4.2% of individuals, XD—4.2% of individuals, DY—12.5% of individuals, and CS—21.4% of individuals) contained *S. symbiotica*. For *H. defensa*, the highest frequency in *R. padi* was 37.5% of HBD population, whereas this bacterium was detected in most populations of *R. maidis* and the highest frequency was 58.3% of XX population. All samples of *R. padi* collected from ZJK were infected with *R. insecticola* and the highest frequency of this bacterium in *R. maidis* was 79.2% of XX population. As for *Rickettsia*, all samples of HEB, KS, DN, and SB populations in *R. padi* were infected, whereas JN population of *R. maidis* had highest infection frequency of 83.3%. The highest frequencies of *Spiroplasma* in *R. padi* and *R. maidis* were 75.0% of AZ and 83.3% of MZ, respectively. There were nine populations (HEB, SY, TL, ZY, LF, XX, YN, KS, and QT) of *R. padi* with frequencies from 5.0% to 20.8% and 11 populations (HEB, GZL, SY, TL, ZY, TS, LF, DZ, LY, LH, and BJ) of *R. maidis* with frequencies from 3.8% to 47.4% had been detected with the infection of *Wolbachia*. Regarding to *Arsenophonus*, the highest frequency in *R. padi* was 85.7% of DN population and there was only one population (DZ) free of *Arsenophonus* in *R. maidis*, the highest frequency in *R. maidis* was 85.7% of CS population.

### 3.3 Comparison of symbiont infection between *R. maidis* and *R. padi* from 19 common locations

**Figure 4:**
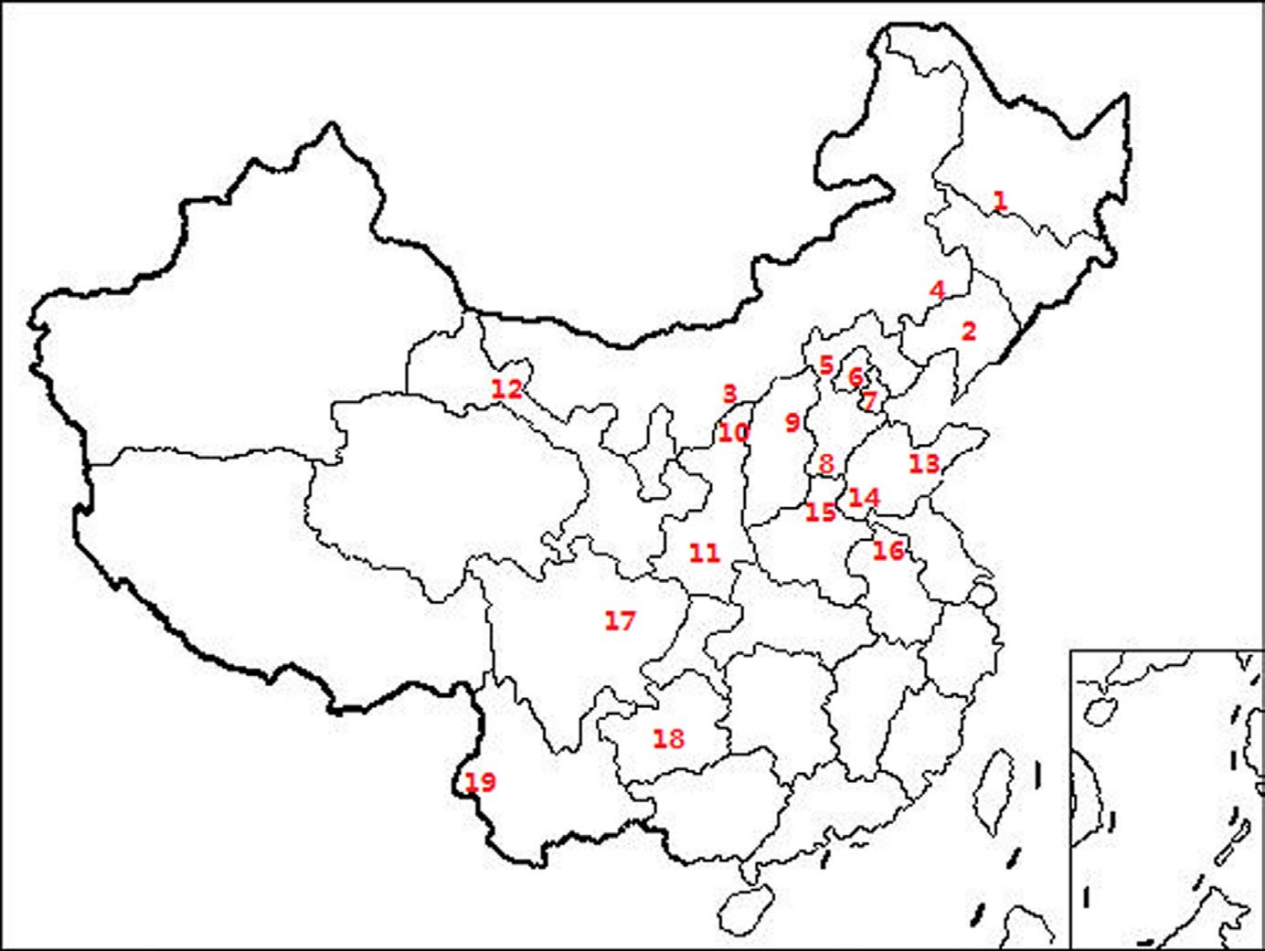
Locations from where both *Rhopalosiphum maids* and *R. padi* were collected. Numbers on the map refers to populations: 1 HEB, 2 SY, 3 TMT, 4 TL, 5 ZJK, 6 BJ, 7 LF, 8 HD, 9 XZ, 10 YuL, 11 YaL, 12 ZY, 13 WF, 14 JNi, 15 XX, 16 SZ, 17 MZ, 18 GY, 19 MS

The infection frequencies of each symbiont within 456 samples of *R. maidis* and 370 samples of *R. padi* from 19 common locations (Figure 4) were compared using the method of Fisher's exact test (Table 3). Frequencies of *H. defensa* (16.0%), *Spiroplasma* (41.4%), and *Arsenophonus* (24.8%) in *R. maidis* exhibited higher prevalence than in *R. padi* (5.9%, 23.5%, and 12.2%, respectively). Conversely, *R. padi* harbored more *R. insecticola* (42.7%) and *Rickettsia* (59.7%) compared with *R. maidis* (34.2% and 51.1%). There was no significant difference of *S. symbiotica* and *Wolbachia* between the two aphid species from 19 common locations.

**Table 3:** Significance of difference of symbiont frequencies between *Rhopalosiphum maidis* and *Rhopalosiphum padi* from 19 common locations

Aphid species pairwise comparison	Symbiont	Fisher's exact test two‐tailed *p*‐values
*R. maidis*/*R. padi*	*Serratia symbiotica*	1.000
*R. maidis*/*R. padi*	*Hamiltonella defensa*	<0.001^a^
*R. padi*/*R. maidis*	*Regiella insecticola*	0.014^a^
*R. padi*/*R. maidis*	*Rickettsia*	0.014^a^
*R. maidis*/*R. padi*	*Spiroplasma*	<0.001^a^
*R. padi*/*R. maidis*	*Wolbachia*	0.861
*R. maidis*/*R. padi*	*Arsenophonus*	<0.001^a^

*Notes*

These are the results of the statistical analysis which was carried out.

^a^Means that there is significant difference of the symbiont frequencies between two aphid species. The aphid species with higher average frequency is listed in the front.

**Figure 5:**
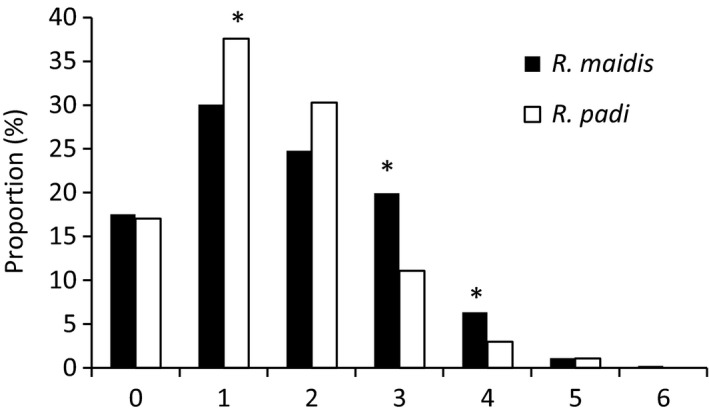
Proportion of symbiont numbers infected in a single aphid of *Rhopalosiphum padi* and *R. maidis* from 19 common locations

The aphids infected with only one symbiont occupied the highest proportion from the 19 sites for both species (Figure 5). However, the proportion of *R. padi* infected with single symbiont (37.6%) was significantly higher than that of *R. maidis* (30.0%) (*p *=* *0.026). Significant higher proportions of *R. maidis* harbored three (20.0%) (*p *=* *0.001) and four (6.4%) (*p *=* *0.033) symbionts per aphid than that of *R. padi* (11.1% and 3.0%). No significant difference (*p > *0.05) was observed between *R. maidis* and *R. padi* of the aphid free of detected symbionts or infected with two, five and six kinds of the symbionts per aphid.

### 3.4 Symbiont infection difference between China and Europe of *R. padi*

The infection frequencies of each symbiont in *R. padi* between 516 samples from China and 69 samples from four European countries were compared using the method of Fisher's exact test (Table 4). The proportions of *H. defensa* (30.4%), *Rickettsia* (76.8%), and *Arsenophonus* (47.8%) in samples collected from Europe were significantly higher than from China (8.1%, 58.7%, and 14.0%, respectively). As for the other symbionts detected in this study, no significant difference was found between Chinese and European samples.

**Table 4:** Significance of difference of symbiont frequencies of *Rhopalosiphum padi* between China and Europe

Aphid groups pairwise comparison	Symbiont	Fisher's exact test two‐tailed *p*‐values
China/Europe	*Serratia symbiotica*	1.000
Europe/China	*Hamiltonella defensa*	<0.001^a^
Europe/China	*Regiella insecticola*	0.605
Europe/China	*Rickettsia*	0.004^a^
Europe/China	*Spiroplasma*	0.109
China/Europe	*Wolbachia*	0.150
Europe/China	*Arsenophonus*	<0.001^a^

*Notes*

These are the results of the statistical analysis which was carried out.

^a^Means there is significant difference of the symbiont frequencies between two aphid groups. The group with higher average frequency is listed in the front.

**Figure 6:**
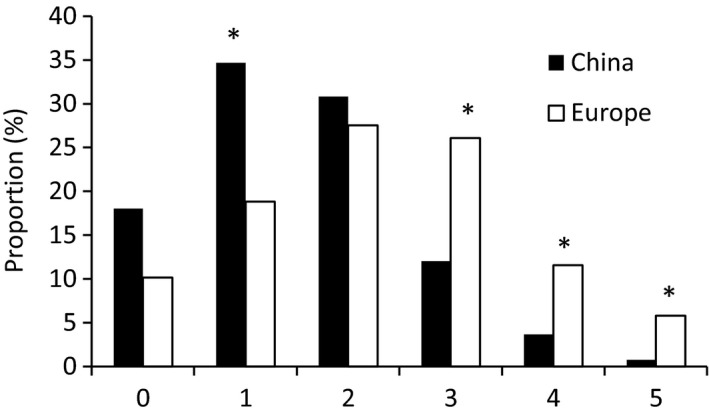
Proportion of symbiont numbers infected in a single *Rhopalosiphum padi* from China and Europe

*R. padi* infected with single symbiont occupied the highest proportion of 34.7% from China and was significantly higher than the proportion from Europe of 18.8% (*p *=* *0.009), however, double‐infected *R. padi* numbers ranked the first among European samples that reached 27.5% (Figure 6). Also, significant higher proportion of European samples harbored 3 (26.1%) (*p *=* *0.004), 4 (11.6%) (*p *=* *0.009), and 5 (5.8%) (*p *=* *0.009) symbionts simultaneously in a single *R. padi* than Chinese samples (12.0%, 3.7%, and 0.8%, respectively). In total 71.0% of European samples were multi‐infected, which was higher than Chinese populations of 47.3%. There was no significant difference between Chinese and European samples that were free of symbionts or double infected (*p > *0.05).

### 3.5 Geographic distribution of facultative symbionts

*H. defensa* was more widely distributed in *R. maidis* (34 of 37 populations were infected) than in *R. padi* (16 of 32 populations were infected). Also, all *H. defensa*‐infected populations with the infection frequencies higher than 10.0% in *R. padi* were collected from areas where the latitudes are higher than 41°N. Furthermore, all the locations of *Wolbachia*‐infected populations were in northern China and the southern‐most locations were LH (33°35′N) of *R. maidis* and XX (35°18′N) of *R. padi* from Henan province. Linear correlation analysis was conducted to access the correlation between the infection frequency of each symbiont and the latitude of the collection sites. The infection frequency of *Wolbachia* was positively correlated with the latitude of the collection sites of *R. maidis* (*r *= 0.372; adj‐*R* ^2^ = 0.113; *p *=* *0.023), whereas there existed negative correlation for *Arsenophonus* of *R. maidis* (*r *=* *−0.443; adj‐*R* ^2^ = 0.173; *p *=* *0.006). Positive correlations were also found for *H. defensa* (*r *=* *0.713; adj‐*R* ^2^ = 0.492; *p *<* *0.001) and *Arsenophonus* (*r *=* *0.586; adj‐*R* ^2^ = 0.322; *p *<* *0.001) of *R. padi*. No significant correlation was detected for other situations.

### 3.6 Phylogenetic relationships

**Figure 7:**
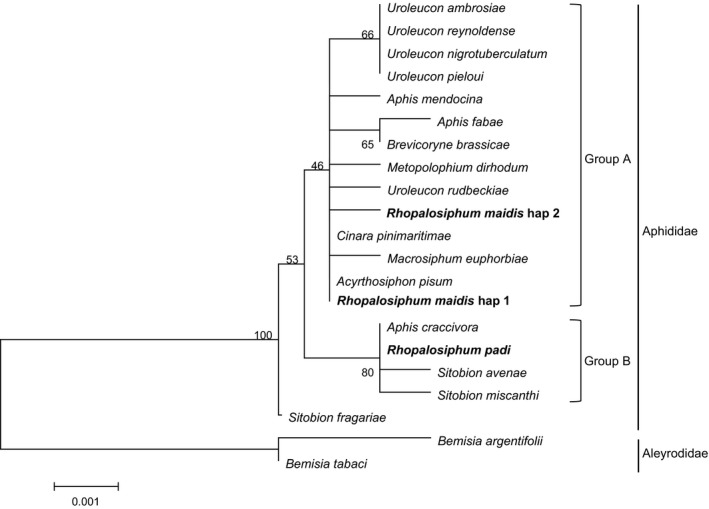
Maximum likelihood phylogenetic analysis inferred from *Hamiltonella defensa* 16S rDNA gene sequences. A bootstrap analysis was carried out and the robustness of each cluster was verified with 1,000 replicates. Values at the cluster branches indicate the results of the bootstrap analysis. Sequences are represented by the names of their host species. The GenBank numbers of the reference sequences are represented in Table A4

A 1,272 bp length fragment of the 16S rDNA sequence of *H. defensa* was obtained after removing the inaccurate terminal sequences. We got one haplotype from 63 infected *R. padi* and two haplotypes from 141 infected *R. maidis* among which, only one *R. maidis* sample of XX population was amplified with the distinct haplotype. The three sequences were deposited in GenBank with accession numbers of KY550361–KY550363. Three haplotype sequences showed 99.8% similarity to the 16S rDNA sequences of *H. defensa* isolated from various insect species. The sequences from the hosts belonging to Aphididae assembled in one cluster, whereas from Aleyrodidae gathered into another cluster. Interestingly, *R. maidis* and *R. padi* are two affinis species that both of them belong to *Rhopalosiphum* genus, with same niche in maize plant in late development stage of maize, however, phylogenetic tree verified that the two haplotypes of *H. defensa* sequences from *R. maidis* fell into group A with the highest homology to *A. pisum* and *Uroleucon rudbeckiae* whereas the haplotype from *R. padi* fell into group B closest to *A. craccivora* (Figure 7).

## 4 DISCUSSION

### 4.1 Frequency of seven facultative symbionts in *R. padi* and *R. maidis*

In this study, we surveyed the infection status of seven facultative symbionts within *R. maidis* and *R. padi* populations collected from maize host. Both *Rhopalosiphum* species exhibited broad symbiotic associations with several facultative symbionts and almost half of the samples (50.8% of *R. maidis* and 50.1% of *R. padi*) were infected with two or more symbionts. In addition, we detected two samples from a number of 882 of *R. maidis* which were superinfected with six facultative symbionts, whereas previous study found that one sample from a number of 318 of *A. pisum* which harbored four facultative symbionts simultaneously (Russell et al., 2013). The infection frequencies of detected symbionts in this study ranged from 0.2% to 60.9%, these differences may result from the benefit‐cost balance associated with harboring symbionts (Simon et al., 2007; Vorburger, Ganesanandamoorthy, & Kwiatkowski, 2013). Furthermore, non‐selective factors such as transmission rates, migration, and drift may also affect the frequency and distribution of the symbionts (Oliver et al., 2014). Interestingly, both *R. maidis* and *R. padi* were rarely infected with *S. symbiotica* (Table 1), whereas this bacterium was frequently detected in *A. pisum* (Sepúlveda, Zepeda‐Paulo, Ramírez, Lavandero, & Figueroa, 2017; Tsuchida, Koga, Shibao, Matsumoto, & Fukatsu, 2002) and *Aphis craccivora* (Brady et al., 2014), which supports the result that symbiont combinations are mainly host specific (Fakhour et al., 2018).

Both *R. maidis* and *R. padi* were frequently infected with *Rickettsia* and *R. insecticola*, whereas previous study demonstrated that *A. pisum* both from pea and alfalfa were rarely infected with *R. insecticola* (Sepúlveda et al., 2017) and both symbionts showed a low frequency in *A. craccivora* from several host plants (Brady et al., 2014). In addition, European samples exhibited significantly higher frequencies of *H. defensa* than Chinese ones although Henry et al. (2015) found *R. padi* collected from UK harbored none symbionts of *R. insecticola*,* H. defensa* as well as *S. symbiotica*. Furthermore, *R. padi* collected from Western Europe were free‐infected with four targeted facultative symbionts (Desneux et al., 2018) whereas in other cases, European *R. padi* lines were found infections with *S. symiotica* (De la Peña et al., 2014) and *H. defensa* (Leybourne et al., 2018). Also, research showed that *Spiroplasma* in *A. pisum* was rarely coinfected with other symbionts (Rock et al., 2017) whereas in our study, this bacterium was relative prevalent in both *R. maidis* and *R. padi* commonly coexisted with other symbionts. Moreover, infection frequencies of symbionts can also differ among host plants species. For instance, *H. defensa* was exclusively detected in *A. craccivora* collected from alfalfa (Brady et al., 2014) and there existed great diversity for the symbionts like *R. insecticola* in *A. pisum* collected from different host plants (Russell et al., 2013).

It is widely accepted that infection frequency and retention of an endosymbiont in insect are determined mainly by three aspects: first, the fidelity of maternal transmission (Luan et al., 2016, 2018); second, influences on the fitness of the insect host; third, the frequency of horizontal transmission (Fukatsu, Nikoh, Kawai, & Koga, 2000; Fukatsu, Tsuchida, Nikoh, & Koga, 2001). The infection frequencies between *R. maidis* and *R. padi* from 19 common collecting sites (Table 3) showed significant difference for five symbionts except for *S*. *symbiotica* and *Wolbachia* which were rarely detected in both aphids. This may result primarily from the fidelity of maternal transmission, whereas horizontal transmission happened occasionally with a low rate (Russell & Moran, 2005). *H. defensa* showed higher prevalence in *R. maidis* than in *R. padi*, as described by Fakhour et al. (2018) that different host species could exhibit different symbiont combinations. Furthermore, significant difference of infection frequencies can be found even from different genotypes of the same aphid species (Zepeda‐Paulo, Villegas, & Lavandero, 2017). A higher proportion of European *R. padi* harbored three, four and five symbionts simultaneously compared with Chinese samples indicating that the infection frequency of facultative symbionts may differ significantly between distant geographical regions. The abiotic factors such as temperature, humidity, day‐length, and rainfall intensity are different between the European and Chinese sampling sites which could affect the infection situation (Tsuchida et al., 2002). For example, the frequency of *S. symbiotica* in *A. pisum* increased in two‐thirds with increasing seasonal temperature in California (Montllor et al., 2002). Moreover, frequencies of symbionts with protective functions may also shift according to the changing of biotic factors such as parasitoid pressures (Smith et al., 2015).

### 4.2 Geographic distribution of facultative symbionts

*Wolbachia* has been detected in *R. maidis* and *R. padi* with low frequencies of 2.8% and 3.3%, respectively. Also, this bacterium was distributed in northern China (above Henan province) and absent in *R. padi* collected from Europe, however, it has been found in other aphids from southern Europe (Greece, Portugal, Spain) (Gómez‐Valero et al., 2004), Iran and Israel (Augustinos et al., 2011), China (Wang, Su, Wen, Jiang, & Qiao, 2014), USA (Russell et al., 2013), and Africa countries (De Clerck et al., 2014). The linear correlation analysis demonstrated that the frequencies of *Wolbachia* in *R. maidis*,* H. defensa* in *R. padi,* and *Arsenophonus* in both *R. maidis* and *R. padi* were correlated with the latitude of collecting locations to some degree. A recent study found that high altitudes act negatively on bacterial communities abundance (Fakhour et al., 2018) and China exhibits diverse ambient conditions from south to north of the latitude ranging from 4°N to 53°N which may affect the symbiont frequency but need further study to verify.

### 4.3 Frequency and phylogenetic analysis of *H. defensa*

Among the tested aphids, 10.8% of *R. padi* and 16.0% *R. maidis* were infected with *H. defensa*, the presence of this symbiotic bacterium could be related with its potential effect on parasitoid wasp defense in aphid host (Cayetano & Vorburger, 2015; Leybourne et al., 2018; Oliver et al., 2005). What's more, host–parasitoid coevolution could be modified by the presence of *H. defensa* (Vorburger, 2014). The infection frequency of *H. defensa* in aphids is affected by transmission efficiency, cost of infection as well as protection against parasitoids (Oliver et al., 2014; Vorburger, 2014). For instance, a longevity cost of harboring *H. defensa* was demonstrated in *A. fabae* (Vorburger & Gouskov, 2011). In our study, only one haplotype was obtained from *R. padi* and two haplotypes from *R. maidis* indicating the high conservation of this genotype (Telesnicki, Ghersa, Martínez‐Ghersa, & Arneodo, 2012). As the phylogenetic tree illustrated, haplotype 1 of *H. defensa* in *R. maidis* could have diverged earlier than haplotype 2 and that *R. maidis* and *R. padi* may acquire *H. defensa* independently on different occasions (Russell et al., 2013). Little transfer of *H. defensa* between *R. maidis* and *R. padi* has occurred yet although a shared feeding niche (West, Cook, Werren, & Godfray, 1998). Our results demonstrated that the two *Rhopalosiphum* species were infected by different *H. defensa* strains which may be determined by host × symbiont genotype interactions (Vorburger & Gouskov, 2011). Furthermore, genotype × genotype interactions exhibited among aphid, symbiont, and parasitoid which could play important role in their coevolution (Vorburger, 2014; Vorburger & Gouskov, 2011; Vorburger, Sandrock, Gouskov, Castañeda, & Ferrari, 2009).

## 5 CONCLUSION

To conclude, both *R. maidis* and *R. padi* presented wide symbiotic relationship with the detected symbionts especially *R. insecticola* and *Rickettsia*, whereas these two *Rhopalosiphum* species were rarely infected with *S. symbiotica*. We hypothesize that the low infection frequency of *S. symbiotica* may be related to the environmental temperature of the collecting regions since *S. symbiotica* has been demonstrated to confer heat tolerance in aphid (Chen, Montllor, & Purcell, 2000; Montllor et al., 2002; Russell & Moran, 2006) which could be tested in the future. Multiple infections were common in these two aphid species, however, single or double infection occupy the highest frequencies. Linear correlation analysis showed the infection frequency of *H. defensa*,* Wolbachia,* and *Arsenophonus* were correlated with the latitude of the collection sites to some extent. The proportions of *H. defensa*,* Rickettsia,* and *Arsenophonus* in European samples were significantly higher than from Chinese ones, which need further investigation to figure out whether it is caused by the environmental factors. In our study, two *Rhopalosiphum* aphid species were collected from the same host plant and over the same period of time which allowed us to compare and contrast their symbiont communities between different geographical locations. Additionally, further work is required to detect the phylogenetic relationship of other symbionts except for *H. defensa* and figure out the symbiont‐mediated adaptation for these aphid species to local conditions which can facilitate insect pest control programs.

## CONFLICT OF INTERESTS

The authors declare that they have no competing interests.

## AUTHORS CONTRIBUTION

J.G. mainly carried out experiment, analyzed the data and was the primary composer of the manuscript; X.L. helped with a part of experiment; N.P. helped with optimizing PCR reaction conditions; K.H. assisted in experiment design; F.F. and Z.W. involved with experiment design and provided supervision. All authors contributed and agreed on the content of the final version.

## ETHICS STATEMENT

None required.

## Data Availability

All data are available in the results section of this paper apart from the three fragments of the 16S rDNA sequence of *H. defensa* which were deposited at www.ncbi.nlm.nih.gov/genbank/ with accession numbers of KY550361, KY550362, and KY550363.

## APPENDIX 1

### 1.1

**Table A1:** Collecting information of *Rhopalosiphum padi* samples investigated

Corn region	Province	Index	Population	Locality	Geo‐coordinates	Date	Number
China	Heilongjiang	1	HEB	Harbin	45°49′N, 126°48′E	August 14, 2014	14
		2	HBP	Harbin	45°38′N, 126°38′E	July 24, 2016	20
		3	HBD	Harbin	45°50′N, 126°50′E	July 24, 2016	16
		4	HG	Hegang	47°8′N, 130°17′E	August 5, 2014	17
		5	SYS	Shuangyashan	46°46′N, 131°06′E	August 7, 2014	3
	Jilin	6	TH	Tonghua	42°22′N, 125°25′E	August 1, 2014	24
	Liaoning	7	SY	Shenyang	41°49′N, 123°33′E	July 28, 2014	24
	Inner	8	TL	Tongliao	43°40′N, 122°21′E	August 11, 2014	24
	Mongolia	9	TMT	Tumd Right Banner	40°36′N, 110°34′E	September 4, 2016	24
	Ningxia	10	QTX	Qingtongxia	38°1′N, 106°4′E	August 4, 2016	24
	Gansu	11	ZY	Zhangye	38°56′N, 100°27′E	August 30, 2016	20
	Hebei	12	ZJK	Zhangjiakou	40°44′N, 114°52′E	August 21, 2014	24
	Shanxi	13	XZ	Xinzhou	38°25′N, 112°43′E	August 26, 2014	24
	Shaanxi	14	YuL	Yulin	38°20′N, 109°46′E	August 28, 2014	24
		15	YaL	Yangling	34°16′N, 108°3′E	September 23, 2014	11
	Hebei	16	HD	Handan	36°56′N, 114°52′E	August 27, 2014	24
		17	LF	Langfang	39°28′N, 116°38′E	August 31, 2014	24
	Shandong	18	JN	Jining	35°5′N, 116°34′E	September 4, 2014	12
		19	WF	Weifang	36°54′6 N, 119°10′E	September 3, 2014	11
	Henan	20	XX	Xinxiang	35°18′N, 113°53′E	September 15, 2014	8
	Beijing	21	BJ	Beijing	40°1′N, 116°16′E	August 19, 2014	23
	Anhui	22	SZ	Suzhou	33°38′N, 117°4′E	September 19, 2014	24
	Yunnan	23	MS	Mangshi	24°26′N, 98°35′E	August 24, 2014	21
	Sichuan	24	MZ	Mianzhu	31°24′N, 104°18′E	July 5, 2016	14
	Guizhou	25	GY	Guiyang	26°30′N, 106°39′E	August 8, 2016	20
	Xinjiang	26	YN	Yining	43°59′N, 81°32′E	August 14, 2014	18
		27	KS	Kashi	39°28′N, 75°59′E	August 14, 2014	6
		28	QT	Qitai	44°4′N, 89°44′E	August 26, 2016	18
Belgium	Namur	29	DN	Dinant	50°34′N, 4°41′E	September 26, 2015	14
Luxembourg	Hesperingen	30	LSB	Alzingen	49°34′N, 6°9′E	September 28, 2015	4
France	Bas‐Rhin	31	FR	Strasbourg	48°38′N, 7°37′E	October 2, 2015	27
Germany	Bayern	32	GM	Ingolstadt	48°44′N, 11°25′E	October 4, 2015	24

**Table A2:** Collecting information of *Rhopalosiphum maidis* samples investigated

Corn region	Province	Index	Population	Locality	Geo‐coordinates	Date	Number
China	Heilongjiang	1	HEB	Harbin	45°49′N, 126°48′E	August 16, 2014	24
	Jilin	2	GZL	Gongzhuling	43°31′N, 124°48′E	September 4, 2014	26
	Liaoning	3	SY	Shenyang	41°49′N, 123°33′E	August 28, 2014	24
	Inner Mongolia	4	TL	Tongliao	43°40′N, 122°21′E	August 11, 2014	19
		5	TMT	Tumd Right Banner	40°36′N, 110°34′E	September 4, 2016	11
	Hebei	6	ZJK	Zhangjiakou	40°45′N, 114°53′E	August 21, 2014	24
		7	LP	Luanping	40°56′N, 117°19′E	September 4, 2015	22
	Shanxi	8	XZ	Xinzhou	38°25′N, 112°44′E	August 26, 2014	24
Shaanxi	9	YuL	Yulin	38°20′N, 109°46′E	August 28, 2014	31
	10	YaL	Yangling	34°17′N, 108°3′E	September 23, 2014	24
Gansu	11	ZY	Zhangye	38°51′N, 100°34′E	August 24, 2014	21
	12	TS	Tianshui	34°44′N, 105°20′E	September 2, 2016	24
	13	PL	Pingliang	35°20′N, 107°22′E	August 24, 2016	10
Hebei	14	HS	Hengshui	37°43′N, 115°44′E	August 20, 2014	24
	15	HD	Handan	36°56′N, 114°52′E	August 27, 2014	19
	16	LC	Luancheng	37°54′N, 114°37′E	August 28, 2014	24
	17	LF	Langfang	39°28′N, 116°38′E	August 31, 2014	27
Shandong	18	JN	Jining	35°5′N, 116°34′E	September 4, 2014	24
	19	DZ	Dezhou	37°28′N, 116°19′E	August 25, 2014	12
	20	WF	Weifang	36°54′N, 119°10′E	September 3, 2014	24
	21	ZQ	Zhangqiu	36°46′N, 117°31′E	September 5, 2014	22
Henan	22	LY	Luoyang	34°38′N, 112°29′E	September 22, 2014	26
	23	XX	Xinxiang	35°18′N, 113°53′E	September 15, 2014	24
	24	LH	Luohe	33°35′N, 114°1′E	September 17, 2014	24
Beijing	25	BJ	Beijing	40°2′N, 116°16′E	August 6, 2014	24
Anhui	26	SZ	Suzhou	33°38′N, 117°4′E	September 19, 2014	24
	27	HF	Hefei	30°24′N, 116°59′E	October 17, 2014	31
Sichuan	28	XD	Xindu	30°47′N, 104°13′E	August 12, 2014	24
	29	MZ	Mianzhu	31°24′N, 104°18′E	August 28, 2014	24
	30	NC	Nanchong	30°53′N, 106°3′E	August 3, 2016	22
Chongqing	31	CQ	Chongqing	29°29′N, 106°22′E	August 6, 2016	34
Guizhou	32	GY	Guiyang	26°30′N, 106°39′E	August 8, 2016	32
Yunnan	33	MS	Mangshi	24°26′N, 98°35′E	September 20, 2014	32
Zhejiang	34	DY	Dongyang	29°27′N, 120°32′E	September 20, 2014	24
Hunan	35	CS	Changsha	28°12′N, 113°05′E	September 14, 2014	14
Guangdong	36	GZ	Guangzhou	23°09′N, 113°21′E	November 8, 2014	34
Hainan	37	YC	Yacheng	18°24′N, 109°12′E	January 17, 2016	29

**Table A3:** PCR primers used in this study

Symbionts	Primer name	Primer sequence (5′–3′)	Product size (bp)	References
*Serratia symbiotica*	16SA1	AGAGTTTGATCMTGGCTCAG	480	(1)
	PASScmp	GCAATGTCTTATTAACACAT		(2)
*Hamiltonella defensa*	PABSF	AGCACAGTTTACTGAGTTCA	1,660	(3)
	16SB1	TACGGYTACCTTGTTACGACTT		(1)
*Regiella insecticola*	U99F	ATCGGGGAGTAGCTTGCTAC	200	(4)
	16SB4	CTAGAGATCGTCGCCTAGGTA		(5)
*Rickettsia*	16SA1	AGAGTTTGATCMTGGCTCAG	200	(1)
	Rick16SR	CATCCATCAGCGATAAATCTTTC		(6)
*Spiroplasma*	16SA1	AGAGTTTGATCMTGGCTCAG	510	(1)
	TKSSspR	TAGCCGTGGCTTTCTGGTAA		(2)
*Wolbachia*	81F	TGGTCCAATAAGTGATGAAGAAAC	610	(7)
	691R	AAAAATTAAACGCTACTCCA		(7)
*Arsenophonus*	16SA1	AGAGTTTGATCMTGGCTCAG	960	(1)
	Ars16SR	TTAGCTCCGGAGGCCACAGT		(5)

**Table A4:** GenBank numbers of the reference sequences

Gene	GenBank accession	Host species
*Hamiltonella defensa*	AB780465	*Acyrthosiphon pisum*
*H. defensa*	AY136136	*Aphis craccivora*
*H. defensa*	KM375938	*Aphis fabae*
*H. defensa*	KF835615	*Aphis mendocina*
*H. defensa*	AY264675	*Bemisia argentifolii*
*H. defensa*	AF400475	*Bemisia tabaci*
*H. defensa*	KT336571	*Brevicoryne brassicae*
*H. defensa*	EU348313	*Cinara pinimaritimae*
*H. defensa*	AY136148	*Macrosiphum euphorbiae*
*H. defensa*	JQ293090	*Metopolophium dirhodum*
*H. defensa*	JX533645	*Sitobion avenae*
*H. defensa*	KM375935	*Sitobion fragariae*
*H. defensa*	HM156641	*Sitobion miscanthi*
*H. defensa*	AF293622	*Uroleucon ambrosiae*
*H. defensa*	AY136162	*Uroleucon nigrotuberculatum*
*H. defensa*	AY136163	*Uroleucon pieloui*
*H. defensa*	AY136164	*Uroleucon reynoldense*
*H. defensa*	AY136166	*Uroleucon rudbeckiae*

## References

[mbo3817-bib-0001] Asplen, M. K. , Bano, N. , Brady, C. M. , Desneux, N. , Hopper, K. R. , Malouines, C. , … Heimpel, G. E. (2014). Specialisation of bacterial endosymbionts that protect aphids from parasitoids. Ecological Entomology, 39, 736–739. 10.1111/een.12153

[mbo3817-bib-0002] Augustinos, A. A. , Santos‐Garcia, D. , Dionyssopoulou, E. , Moreira, M. , Papapanagiotou, A. , Scarvelakis, M. , … Borges, P. A. (2011). Detection and characterization of *Wolbachia* infections in natural populations of aphids: Is the hidden diversity fully unraveled? PLoS ONE, 6, 1–10. 10.1371/journal.pone.0028695 PMC3236762 22174869

[mbo3817-bib-0003] Baumann, P. (2005). Biology of bacteriocyte‐associated endosymbionts of plant sap‐sucking insects. Annual Review of Microbiology, 59, 155–189. 10.1146/annurev.micro.59.030804.121041 16153167

[mbo3817-bib-0004] Brady, C. M. , Asplen, M. K. , Desneux, N. , Heimpel, G. E. , Hopper, K. R. , Linnen, C. R. , … White, J. A. (2014). Worldwide populations of the aphid *Aphis craccivora* are infected with diverse facultative bacterial symbionts. Microbial Ecology, 67, 195–204. 10.1007/s00248-013-0314-0 24233285

[mbo3817-bib-0005] Castañeda, L. E. , Sandrock, C. , & Vorburger, C. (2010). Variation and covariation of life history traits in aphids are related to infection with the facultative bacterial endosymbiont *Hamiltonella defensa* . Biological Journal of the Linnean Society, 100, 237–247. 10.1111/j.1095-8312.2010.01416.x

[mbo3817-bib-0006] Cayetano, L. , & Vorburger, C. (2015). Symbiont‐conferred protection against Hymenopteran parasitoids in aphids: How general is it? Ecological Entomology, 40, 85–93. 10.1111/een.12161

[mbo3817-bib-0007] Chen, Y. T. , Guo, M. K. , Zhu, X. Y. , Gao, W. D. , Da, F. C. , & Li, L. (1996). Evaluation of resistance to germplasm dwarf mosaic virus in maize. Plant Protection, 22, 13–15.

[mbo3817-bib-0008] Chen, D.‐Q. , Montllor, C. B. , & Purcell, A. H. (2000). Fitness effects of two facultative endosymbiotic bacteria on the pea aphid, *Acyrthosiphon pisum*, and the blue alfalfa aphid, *A. kondoi* . Entomologia Experimentalis et Applicata, 95, 315–323. 10.1046/j.1570-7458.2000.00670.x

[mbo3817-bib-0009] De Clerck, C. , Fujiwara, A. , Joncour, P. , Léonard, S. , Félix, M.‐L. , Francis, F. , … Massart, S. (2015). A metagenomic approach from aphid's hemolymph sheds light on the potential roles of co‐existing endosymbionts. Microbiome, 3(1), 1–11. 26667400 10.1186/s40168-015-0130-5 PMC4678535

[mbo3817-bib-0010] De Clerck, C. , Tsuchida, T. , Massart, S. , Lepoivre, P. , Francis, F. , & Jijakli, M. H. (2014). Combination of genomic and proteomic approaches to characterize the symbiotic population of the banana aphid (Hemiptera: Aphididae). Environmental Entomology, 43(1), 29–36. 10.1603/EN13107 24472200

[mbo3817-bib-0011] De la Peña, E. , Vandomme, V. , & Frago, E. (2014). Facultative endosymbionts of aphid populations from coastal dunes of the North Sea. Belgian Journal of Zoology, 144(1), 41–50.

[mbo3817-bib-0012] Desneux, N. , Asplen, M. K. , Brady, C. M. , Heimpel, G. E. , Hopper, K. R. , Luo, C. , … White, J. A. (2018). Intraspecific variation in facultative symbiont infection among native and exotic pest populations: Potential implications for biological control. Biological Control, 116, 27–35. 10.1016/j.biocontrol.2017.06.007

[mbo3817-bib-0013] Douglas, A. (1998). Nutritional interactions in insect‐microbial symbioses: Aphids and their symbiotic bacteria Buchnera. Annual Review of Entomology, 43(1), 17–37. 10.1146/annurev.ento.43.1.17 15012383

[mbo3817-bib-0014] Fakhour, S. , Ambroise, J. , Renoz, F. , Foray, V. , Gala, J.‐L. , & Hance, T. (2018). A large‐scale field study of bacterial communities in cereal aphid populations across Morocco. FEMS Microbiology Ecology, 94, fiy003. 10.1093/femsec/fiy003 29346623

[mbo3817-bib-0015] Ferrari, J. , West, J. A. , Via, S. , & Godfray, H. C. J. (2012). Population genetic structure and secondary symbionts in host‐associated populations of the pea aphid complex. Evolution, 66, 375–390. 10.1111/j.1558-5646.2011.01436.x 22276535

[mbo3817-bib-0016] Folmer, O. , Black, M. , Hoeh, W. , Lutz, R. , & Vrijenhoek, R. (1994). DNA primers for amplification of mitochondrial cytochrome c oxidase subunit I from diverse metazoan invertebrates. Molecular Marine Biology and Biotechnology, 3, 294–299. 7881515

[mbo3817-bib-0017] Fukatsu, T. , Nikoh, N. , Kawai, R. , & Koga, R. (2000). The secondary endosymbiotic bacterium of the pea aphid *Acyrthosiphon pisum* (Insecta: Homoptera). Applied and Environmental Microbiology, 66, 2748–2758. 10.1128/AEM.66.7.2748-2758.2000 10877764 PMC92069

[mbo3817-bib-0018] Fukatsu, T. , Tsuchida, T. , Nikoh, N. , & Koga, R. (2001). *Spiroplasma* symbiont of the pea aphid, *Acyrthosiphon pisum* (Insecta: Homoptera). Applied and Environmental Microbiology, 67, 1284–1291. 10.1128/AEM.67.3.1284-1291.2001 11229923 PMC92726

[mbo3817-bib-0019] Gómez‐Valero, L. , Soriano‐Navarro, M. , Pérez‐Brocal, V. , Heddi, A. , Moya, A. , García‐Verdugo, J. M. , & Latorre, A. (2004). Coexistence of *Wolbachia* with *Buchnera aphidicola* and a secondary symbiont in the aphid *Cinara cedri* . Journal of Bacteriology, 186, 6626–6633. 10.1128/JB.186.19.6626-6633.2004 15375144 PMC516615

[mbo3817-bib-0020] Guo, J. Q. , Hatt, S. , He, K. L. , Chen, J. L. , Francis, F. , & Wang, Z. Y. (2017). Nine facultative endosymbionts in aphids. A review. Journal of Asia‐Pacific Entomology, 20, 794–801. 10.1016/j.aspen.2017.03.025

[mbo3817-bib-0021] Henry, L. M. , Maiden, M. C. J. , Ferrari, J. , & Godfray, H. C. J. (2015). Insect life history and the evolution of bacterial mutualism. Ecology Letters, 18, 516–525. 10.1111/ele.12425 25868533

[mbo3817-bib-0022] Laughton, A. M. , Fan, M. H. , & Gerardo, N. M. (2013). The combined effects of bacterial symbionts and ageing on life history traits in the pea aphid, *Acyrthosiphon pisum* . Applied & Environmental Microbiology, 80, 470–477. 24185857 10.1128/AEM.02657-13 PMC3911086

[mbo3817-bib-0023] Leybourne, D. J. , Bos, J. I. B. , Valentine, T. A. , & Karley, A. J. (2018). The price of protection: A defensive endosymbiont impairs nymph growth in the bird cherry‐oat aphid, *Rhopalosiphum padi* . Insect Science, 1–17. 10.1111/1744-7917.12606 PMC7379937 29797656

[mbo3817-bib-0024] Li, T. , Xiao, J.‐H. , Xu, Z.‐H. , Murphy, R. W. , & Huang, D.‐W. (2011). Cellular tropism, population dynamics, host range and taxonomic status of an aphid secondary symbiont, SMLS (*Sitobion miscanthi* L type symbiont). PLoS ONE, 6, e21944 10.1371/journal.pone.0021944 21789197 PMC3137594

[mbo3817-bib-0025] Luan, J. B. , Shan, H. W. , Isermann, P. , Huang, J. H. , Lammerding, J. , Liu, S. S. , & Douglas, A. E. (2016). Cellular and molecular remodeling of a host cell for vertical transmission of bacterial symbionts. Proceedings of the Royal Society B Biological Sciences, 283, 20160580 10.1098/rspb.2016.0580 PMC4936034 27358364

[mbo3817-bib-0026] Luan, J. B. , Sun, X. P. , Fei, Z. , & Douglas, A. E. (2018). Maternal inheritance of a single somatic animal cell displayed by the bacteriocyte in the whitefly *Bemisia tabaci* . Current Biology, 28, 459–465. 10.1016/j.cub.2017.12.041 29395925 PMC5807091

[mbo3817-bib-0027] Łukasik, P. , Dawid, M. , Ferrari, J. , & Godfray, H. C. (2013). The diversity and fitness effects of infection with facultative endosymbionts in the grain aphid, *Sitobion avenae* . Oecologia, 173, 985–996. 10.1007/s00442-013-2660-5 23624672

[mbo3817-bib-0028] Łukasik, P. , van Asch, M. , Guo, H. , Ferrari, J. , & Godfray, H. C. J. (2013). Unrelated facultative endosymbionts protect aphids against a fungal pathogen. Ecology Letters, 16, 214–218. 10.1111/ele.12031 23137173

[mbo3817-bib-0029] Manzano‐Marín, A. , & Latorre, A. (2014). Settling down: The genome of serratia symbiotica from the aphid *Cinara tujafilina* zooms in on the process of accommodation to a cooperative intracellular life. Genome Biology and Evolution, 6, 1683–1698. 10.1093/gbe/evu133 24951564 PMC4122931

[mbo3817-bib-0030] Montllor, C. B. , Maxmen, A. , & Purcell, A. H. (2002). Facultative bacterial endosymbionts benefit pea aphids *Acyrthosiphon pisum* under heat stress. Ecological Entomology, 27, 189–195. 10.1046/j.1365-2311.2002.00393.x

[mbo3817-bib-0031] Oliver, K. M. , Campos, J. , Moran, N. A. , & Hunter, M. S. (2008). Population dynamics of defensive symbionts in aphids. Proceedings of the Royal Society B: Biological Sciences, 275, 293–299. 10.1098/rspb.2007.1192 PMC2593717 18029301

[mbo3817-bib-0032] Oliver, K. M. , Degnan, P. H. , Burke, G. R. , & Moran, N. A. (2010). Facultative symbionts in aphids and the horizontal transfer of ecologically important traits. Annual Review of Entomology, 55, 247–266. 10.1146/annurev-ento-112408-085305 19728837

[mbo3817-bib-0033] Oliver, K. M. , Moran, N. A. , & Hunter, M. S. (2005). Variation in resistance to parasitism in aphids is due to symbionts not host genotype. Proceedings of the National Academy of Sciences of the United States of America, 102, 12795–12800. 10.1073/pnas.0506131102 16120675 PMC1200300

[mbo3817-bib-0034] Oliver, K. M. , Moran, N. A. , & Hunter, M. S. (2006). Costs and benefits of a superinfection of facultative symbionts in aphids. Proceedings of the Royal Society B: Biological Sciences, 273, 1273–1280. 10.1098/rspb.2005.3436 PMC1560284 16720402

[mbo3817-bib-0035] Oliver, K. M. , Russell, J. A. , Moran, N. A. , & Hunter, M. S. (2003). Facultative bacterial symbionts in aphids confer resistance to parasitic wasps. Proceedings of the National Academy of Sciences of the United States of America, 100, 1803–1807. 10.1073/pnas.0335320100 12563031 PMC149914

[mbo3817-bib-0036] Oliver, K. M. , Smith, A. H. , & Russell, J. A. (2014). Defensive symbiosis in the real world—advancing ecological studies of heritable, protective bacteria in aphids and beyond. Functional Ecology, 28, 341–355. 10.1111/1365-2435.12133

[mbo3817-bib-0037] Parry, H. R. , Macfadyen, S. , & Kriticos, D. J. (2012). The geographical distribution of Yellow dwarf viruses and their aphid vectors in Australian grasslands and wheat. Australasian Plant Pathology, 41, 375–387. 10.1007/s13313-012-0133-7

[mbo3817-bib-0038] Polin, S. , Le Gallic, J.‐F. , Simon, J.‐C. , Tsuchida, T. , & Outreman, Y. (2015). Conditional reduction of predation risk associated with a facultative symbiont in an insect. PLoS ONE, 10, e0143728 10.1371/journal.pone.0143728 26618776 PMC4664394

[mbo3817-bib-0039] Rock, D. I. , Smith, A. H. , Joffe, J. , Albertus, A. , Wong, N. , O'Connor, M. , … Russell, J. A. (2017). Context‐dependent vertical transmission shapes strong endosymbiont community structure in the pea aphid, *Acyrthosiphon pisum* . Molecular Ecology, 27, 2039–2056. 29215202 10.1111/mec.14449

[mbo3817-bib-0040] Russell, J. A. , Latorre, A. , Sabater‐Muñoz, B. , Moya, A. , & Moran, N. A. (2003). Side‐stepping secondary symbionts: Widespread horizontal transfer across and beyond the Aphidoidea. Molecular Ecology, 12, 1061–1075. 10.1046/j.1365-294X.2003.01780.x 12753224

[mbo3817-bib-0041] Russell, J. A. , & Moran, N. A. (2005). Horizontal transfer of bacterial symbionts: Heritability and fitness effects in a novel aphid host. Applied and Environmental Microbiology, 71, 7987–7994. 10.1128/AEM.71.12.7987-7994.2005 16332777 PMC1317397

[mbo3817-bib-0042] Russell, J. A. , & Moran, N. A. (2006). Costs and benefits of symbiont infection in aphids: Variation among symbionts and across temperatures. Proceedings of the Royal Society B: Biological Sciences, 273, 603–610. 10.1098/rspb.2005.3348 PMC1560055 16537132

[mbo3817-bib-0043] Russell, J. A. , Weldon, S. , Smith, A. H. , Kim, K. L. , Hu, Y. , Łukasik, P. , … Oliver, K. M. (2013). Uncovering symbiont‐driven genetic diversity across North American pea aphids. Molecular Ecology, 22, 2045–2059. 10.1111/mec.12211 23379399

[mbo3817-bib-0044] Saksena, K. N. , Singh, S. R. , & Sill, Jr., W. H. (1964). Transmission of Barley yellow‐dwarf virus by four biotypes of the corn leaf aphid, *Rhopalosiphum maidis* . Journal of Economic Entomology, 57, 569–571. 10.1093/jee/57.4.569

[mbo3817-bib-0045] Sandström, J. P. , Russell, J. A. , White, J. P. , & Moran, N. A. (2001). Independent origins and horizontal transfer of bacterial symbionts of aphids. Molecular Ecology, 10, 217–228. 10.1046/j.1365-294X.2001.01189.x 11251800

[mbo3817-bib-0046] Scarborough, C. L. , Ferrari, J. , & Godfray, H. C. J. (2005). Aphid protected from pathogen by endosymbiont. Science, 310, 1781 10.1126/science.1120180 16357252

[mbo3817-bib-0047] Sepúlveda, D. A. , Zepeda‐Paulo, F. , Ramírez, C. C. , Lavandero, B. , & Figueroa, C. C. (2017). Diversity, frequency and geographic distribution of facultative bacterial endosymbionts in introduced aphid pests. Insect Science, 24, 511–521. 10.1111/1744-7917.12313 26773849

[mbo3817-bib-0048] Simon, J. C. , Sakurai, M. , Bonhomme, J. , Tsuchida, T. , Koga, R. , & Fukatsu, T. (2007). Elimination of a specialised facultative symbiont does not affect the reproductive mode of its aphid host. Ecological Entomology, 32, 296–301. 10.1111/j.1365-2311.2007.00868.x

[mbo3817-bib-0049] Smith, A. H. , Łukasik, P. , O'Connor, M. P. , Lee, A. , Mayo, G. , Drott, M. T. , … Messina, A. (2015). Patterns, causes and consequences of defensive microbiome dynamics across multiple scales. Molecular Ecology, 24, 1135–1149. 10.1111/mec.13095 25683348

[mbo3817-bib-0050] Smyrnioudis, I. N. , Harrington, R. , Clark, S. J. , & Katis, N. (2001). The effect of natural enemies on the spread of Barley yellow dwarf virus (BYDV) by *Rhopalosiphum padi* (Hemiptera: Aphididae). Bulletin of Entomological Research, 91, 301–306. 10.1079/BER2001110 11587627

[mbo3817-bib-0051] Stamatakis, A. , Hoover, P. , & Rougemont, J. (2008). A rapid bootstrap algorithm for the RAxML Web servers. Systematic Biology, 57, 758–771. 10.1080/10635150802429642 18853362

[mbo3817-bib-0052] Sunnucks, P. , & Hales, D. F. (1996). Numerous transposed sequences of mitochondrial cytochrome oxidase I‐II in aphids of the genus *Sitobion* (Hemiptera: Aphididae). Molecular Biology and Evolution, 13, 510–524. 10.1093/oxfordjournals.molbev.a025612 8742640

[mbo3817-bib-0053] Telesnicki, M. C. , Ghersa, C. M. , Martínez‐Ghersa, M. A. , & Arneodo, J. D. (2012). Molecular identification of the secondary endosymbiont *Hamiltonella defensa* in the rose‐grain aphid *Metopolophium dirhodum* . Revista Argentina de Microbiologia, 44, 255–258. 23267621

[mbo3817-bib-0054] Tsuchida, T. , Koga, R. , Shibao, H. , Matsumoto, T. , & Fukatsu, T. (2002). Diversity and geographic distribution of secondary endosymbiotic bacteria in natural populations of the pea aphid, *Acyrthosiphon pisum* . Molecular Ecology, 11, 2123–2135. 10.1046/j.1365-294X.2002.01606.x 12296954

[mbo3817-bib-0055] Unckless, R. L. , & Jaenike, J. (2012). Maintenance of a male‐killing *wolbachia* in *Drosophila innubila* by male‐killing dependent and male‐killing independent mechanisms. Evolution, 66, 678–689. 10.1111/j.1558-5646.2011.01485.x 22380432

[mbo3817-bib-0056] Vorburger, C. (2014). The evolutionary ecology of symbiont‐conferred resistance to parasitoids in aphids. Insect Science, 21, 251–264. 24167113 10.1111/1744-7917.12067

[mbo3817-bib-0057] Vorburger, C. , Ganesanandamoorthy, P. , & Kwiatkowski, M. (2013). Comparing constitutive and induced costs of symbiont‐conferred resistance to parasitoids in aphids. Ecology & Evolution, 3, 706–713. 10.1002/ece3.491 23533102 PMC3605857

[mbo3817-bib-0058] Vorburger, C. , & Gouskov, A. (2011). Only helpful when required: A longevity cost of harbouring defensive symbionts. Journal of Evolutionary Biology, 24, 1611–1617. 10.1111/j.1420-9101.2011.02292.x 21569156

[mbo3817-bib-0059] Vorburger, C. , Sandrock, C. , Gouskov, A. , Castañeda, L. E. , & Ferrari, J. (2009). Genotypic variation and the role of defensive endosymbionts in an all‐parthenogenetic host‐parasitoid interaction. Evolution, 63, 1439–1450. 10.1111/j.1558-5646.2009.00660.x 19228189

[mbo3817-bib-0060] Wagner, S. M. , Martinez, A. J. , Ruan, Y.‐M. , Kim, K. L. , Lenhart, P. A. , Dehnel, A. C. , … White, J. A. (2015). Facultative endosymbionts mediate dietary breadth in a polyphagous herbivore. Functional Ecology, 29, 1402–1410. 10.1111/1365-2435.12459

[mbo3817-bib-0061] Wang, Z. , Su, X. M. , Wen, J. , Jiang, L. Y. , & Qiao, G. X. (2014). Widespread infection and diverse infection patterns of *Wolbachia* in Chinese aphids. Insect Science, 21, 313–325. 10.1111/1744-7917.12102 24395812

[mbo3817-bib-0062] Watts, T. , Haselkorn, T. S. , Moran, N. A. , & Markow, T. A. (2009). Variable incidence of *Spiroplasma* infections in natural populations of *Drosophila* species. PLoS ONE, 4, e5703 10.1371/journal.pone.0005703 19492088 PMC2683927

[mbo3817-bib-0063] West, S. A. , Cook, J. M. , Werren, J. H. , & Godfray, H. C. J. (1998). *Wolbachia* in two insect host–parasitoid communities. Molecular Ecology, 7, 1457–1465. 10.1046/j.1365-294x.1998.00467.x 9819901

[mbo3817-bib-0064] Zepeda‐Paulo, F. , Ortiz‐Martínez, S. , Silva, A. X. , & Lavandero, B. (2018). Low bacterial community diversity in two introduced aphid pests revealed with 16S rRNA amplicon sequencing. PeerJ, 6, e4725 10.7717/peerj.4725 29761046 PMC5944429

[mbo3817-bib-0065] Zepeda‐Paulo, F. , Villegas, C. , & Lavandero, B. (2017). Host genotype‐endosymbiont associations and their relationship with aphid parasitism at the field level. Ecological Entomology, 42, 95.

[mbo3817-bib-0066] Zytynska, S. E. , & Weisser, W. W. (2016). The natural occurrence of secondary bacterial symbionts in aphids. Ecological Entomology, 41(1), 13–26. 10.1111/een.12281

